# Factors Associated With Nocturia-Related Quality of Life in Men With Lower Urinary Tract Symptoms and Treated With Tamsulosin Oral Controlled Absorption System in a Non-Interventional Study

**DOI:** 10.3389/fphar.2020.00816

**Published:** 2020-06-04

**Authors:** Martin C. Michel, Helmut Schumacher, Ludwig Mehlburger, Jean J.M.C.H. de la Rosette

**Affiliations:** ^1^Department of Pharmacology, Johannes Gutenberg University, Mainz, Germany; ^2^Consultant, Ingelheim, Germany; ^3^Department of Medical Affairs, Boehringer Ingelheim, Ingelheim, Germany; ^4^Department of Urology, Istanbul Medipol University, Istanbul, Turkey

**Keywords:** male lower urinary tract symptoms, nocturia, quality of life, diabetes, heart failure

## Abstract

Nocturia impairs quality of life (QoL). We have performed a non-interventional study in which men with lower urinary tract symptoms (LUTS) were treated for at least 3 months with tamsulosin oral controlled absorption system (0.4 mg q.d.). Other than observing efficacy and tolerability of this drug formulation, the study was designed to explore the relative roles of number of nocturia episodes and of non-urological causes of nocturia on nocturia-related QoL at baseline and treatment-associated changes thereof. The study enrolled 5775 men seeking treatment of their LUTS. Tamsulosin improved LUTS, e.g. International Prostate Symptom Score from 19.5 ± 5.9 to 10.1 ± 4.9 (means ± SD). This was associated by clinically meaningful improvements in the Nocturia QoL score (from 45 ± 19 to 73 ± 17 points) and other QoL scores. Number of nocturnal voids was the key driver of all QoL scores at baseline; change of number of nocturia episodes that of improvement of all QoL scores upon treatment. In contrast, non-urological causes of nocturia such as heart failure, diabetes, sleep apnea, fluid or alcohol intake or use of diuretics or hypnotics had only small if any effects on baseline QoL or treatment-associated improvements thereof. The observed effects of non-urological causes on QoL apparently were largely driven by their effect on number of nocturnal voids. These data further support the idea that improvement of nocturia may be an important treatment goal in male LUTS.

## Introduction

The International Continence Society defines nocturia as “the number of times urine is passed during the main sleep period” (D’ancona et al., 2019). The symptom nocturia can result from various pathophysiological causes including bladder pathologies such as overactivity, fibrosis, and cancer, inappropriate fluid intake (too much or at wrong time), and excessive diuresis; the latter can result from excessive fluid intake, high osmotic load, reduced exposure, or resistance to vasopressin, increased exposure to atrial natriuretic peptide, and/or ingestion of diuretic substances such as diuretics, alcohol, and caffeine (Schneider et al., 2009; Hall et al., 2011). Accordingly, nocturia can be caused by urological conditions such as benign prostatic obstruction, overactive bladder syndrome or (advanced) prostate or bladder cancer and by non-urological conditions, such as heart failure and sleep apnea (both leading to increased secretion of atrial natriuretic peptide), diabetes mellitus (potentially leading to increased diuresis by osmotic load), and nocturnal polyuria and diabetes insipidus (impaired secretion of or responsiveness to vasopressin); it can also be caused by lifestyle factors, including excessive intake of fluid, in general, and of caffeine or alcohol, in particular; finally, enhanced diuresis it can be iatrogenic due to prescription of diuretics (Schneider et al., 2009; Cornu et al., 2012; Bosch and Weiss, 2013). The reported prevalence of nocturia ranges widely depending on setting of data acquisition, gender, age, and applied definition of the symptom, possibly also reflecting the multiplicity of possible causes (Bosch and Weiss, 2013).

Nocturia is medically relevant because it can lead to an increased number of falls and fractures (Temml et al., 2009) and an increased overall mortality (Asplund, 1999; Bursztyn et al., 2006). It is also associated with a greater prevalence of depressive symptoms (Kupelian et al., 2012). Perhaps of even more direct relevance to patients, nocturia is a highly bothersome condition with major adverse impact on quality of life (QoL) (Schneider et al., 2009). In men with lower urinary tract symptoms (LUTS), nocturia is one of the most bothersome symptoms and strongly correlated with disease-specific QoL score (dsQoL) (Ushijima et al., 2006; Seki et al., 2008; Van Dijk et al., 2010; Kupelian et al., 2012). Interestingly, nocturia apparently has little impact on QoL if it occurs only up to once a night, but two or more episodes per night are typically considered bothersome (Tikkinen et al., 2010). Nocturia also reduces the QoL of the partner of the afflicted patient, presumably because the sleep pattern of the partner is also disturbed (Mitroupoulos et al., 2002).

Based on the impact of nocturia on morbidity, mortality, and QoL, various treatments have been explored for their ability to reduce nocturia. Surgery and minimally invasive procedures to reduce prostate size markedly reduced nocturia and other storage symptoms (Yoshimura et al., 2003; Cai et al., 2006; Seki et al., 2008; Van Dijk et al., 2010). While vasopressin receptor agonists reduce nocturia in subjects with nocturnal polyuria, medicines typically used in the treatment of male LUTS such as α_1_-adrenoceptor antagonists or muscarinic receptor antagonists as drug classes caused only small and inconsistent improvements of nocturia compared to placebo in controlled studies (Schneider et al., 2009; Cornu et al., 2012; Xue et al., 2018). However, clinical experience shows a more substantial reduction of nocturia by α_1_-adrenoceptor antagonists in men with LUTS in non-interventional studies (NIS; about 1.3 as compared to ≤0.3 episodes as compared to placebo in controlled studies) (Schneider et al., 2009). It is assumed that α_1_-adrenoceptor antagonists or muscarinic receptor antagonists are not very effective against nocturia as compared to placebo because of the multifactorial pathophysiology underlying the symptom. However, the contribution of nocturia causes in men with urological disease, such as LUTS, has not been explored.

Tamsulosin Oral Controlled Absorption System^®^ (OCAS) is a more recent formulation of tamsulosin (Van Dijk et al., 2006). It is similarly effective as the capsule formulation of tamsulosin (Chapple et al., 2005) but its absorption is not affected by concomitant food intake (Michel et al., 2005a), it has lower peak plasma levels (Michel et al., 2005a) and causes fewer cardiovascular symptoms than the capsule formulation (Michel et al., 2005b; Michel et al., 2005c). After a pilot study had indicated bigger reductions of nocturia episodes than previously reported with the capsule formulation (Djavan et al., 2005), we became interested in possible effects of tamsulosin OCAS on nocturia and related QoL. Therefore, a NIS was designed to explore the tolerability and efficacy of tamsulosin OCAS in men with LUTS with a focus on nocturia and nocturia-associated QoL. The latter was partly addressed by the validated Nocturia QoL score (NQoL) (Abraham et al., 2004; Cai et al., 2006; Chartier-Kastler and Davidson, 2007). An additional aim of the study was to further explore the factors associated with QoL.

## Patients and Methods

An open-label NIS was performed between April 2005 and February 2006 as part of the post-marketing surveillance following the launch of tamsulosin OCAS in Germany under §67, 6 of the German Drug Act. Due to the non-interventional character of the study and the transfer of only anonymous data to the sponsor, neither ethical committee approval nor informed patient consent was required or recommended according to local laws and regulations in Germany at the time the NIS was performed. However, it was recorded with the German Federal Association of Statutory Health Insurance, the Kassenärztliche Bundesvereinigung and the federal regulatory body (Bundesinstitut für Arzneimittel und Medizinprodukte); it was also registered on clinicaltrials.gov (NCT02245542). The study was designed to assess the efficacy and safety of tamsulosin OCAS with an emphasis on nighttime symptoms and associated QoL. Based on its non-interventional character, the study did not have formal inclusion or exclusion criteria other than the summary of product characteristics. Rather participating board-certified, office-based urologists were asked to systematically document their observations in men with LUTS who were to be treated with tamsulosin OCAS (0.4 mg q.d. for at least 3 months) based on the physician’s medical judgment. Planned enrollment was 5,500 patients.

The following parameters were captured at study entry: date of birth, height, body weight, previous male LUTS treatments (new diagnosis, watchful waiting, herbal remedies, 5α-reductase inhibitors, α-blockers, others), comorbidity with congestive heart failure, diabetes mellitus or sleep apnea, and comedication with diuretics or hypnotics; if available, prostate specific antigen (PSA) was also documented. Both at study entry and study end, the following parameters were additionally documented: all items of the International Prostate Symptom Score (IPSS), its associated dsQoL question, amount of fluid intake (categorically as small, < 1 L; medium, 1–2 L; and high fluid intake, > 2 L), and alcohol (categorically as no or yes) on the day before the office visit, and all items of the NQoL; if available Q_max_ and post-voiding residual urine (PVR). Total IPPS, total NQoL, and its two sub-domains, NQoL-sleep and NQoL-bother, were calculated based on the individual items; question 7 of the IPSS was used to capture the number of nocturnal voids. At study end, it was additionally documented whether treatment was completed as planned (yes; no due to lack of efficacy; no due to adverse events (AE); no, lost to follow-up); if AEs had occurred, a specific AE recording form was to be filled with additional information. Moreover, general tolerability was documented as very good, good, satisfactory or poor.

The study protocol and statistical analysis plan were finalized before data analysis started. In line with the non-interventional character of the study, no hypothesis-testing statistical analysis was performed. Rather descriptive data were documented as % of subjects or as means ± SD. We applied general linear models to assess the effects of potential explanatory variables (age, weight, presence of concomitant heart failure, diabetes or sleep apnea, concomitant use of diuretics or hypnotics, fluid and alcohol intake) on the dependent variables of dsQoL, NQoL, and its sub-domains and total IPSS; the effects on Q_max_, PVR, and PSA were assessed for comparison. The models exploring changes of dependent variables after treatment additionally including the baseline value of the dependent variable as explanatory variable and changes (rather than baseline values) of number of nocturnal voids; no model for PSA was run after treatment because α-blockers do not affect PSA. All models were run in parallel with and without number of nocturia episodes as additional explanatory variable. Effect sizes were calculated only if the descriptive p-value for an explanatory variable within a model was < 0.01. The general linear models for the baseline data involved all participants, those for the treatment-associated improvements only those with data at study entry and study end. Calculations were performed using the SAS program package (version 8.2; SAS Institute, Cary, NC, USA). All reported p-values should be interpreted as descriptive only. Overall reporting follows the STROBE guidelines for cohort studies (https://strobe-statement.org).

## Results

### Demographic and Baseline Clinical Data

Data from a total of 5775 men being documented by 822 urology offices were collected. Patients had an average age of 66 ± 9 years, height of 176 ± 6 cm, weight of 83 ± 10 kg, and a body mass index of 26.7 ± 2.8 kg/m^2^. Many participants were newly diagnosed (38.5%), whereas others had previously been followed by watchful waiting (10.3%) or been treated with an herbal remedy (29.7%), an α-blocker (21.5%) or a 5α-reductase inhibitor (3.4%; multiple nominations possible). The comorbidities of heart failure, diabetes or sleep apnea were noted in 15.3%, 21.0% and 1.7% of patients, respectively. Concomitant use of diuretics and hypnotics was reported in 11.4% and 2.6% of patients, respectively. Small (< 1 L), medium (1–2 L), and high fluid intake (> 2 L) was reported in 7.2%, 72.9% and 18.6% of patients. Use of alcoholic beverages on the evening prior to the office visit was reported by 24.0% of patients.

At baseline prior to treatment, patients had an average IPSS of 19.5 ± 5.9 points, a Q_max_ of 11.5 ± 3.7 ml/s, a PVR of 69.6 ± 48.7 ml, and a PSA of 2.6 ± 2.2 ng/ml. The patients had 2.8 ± 1.0 nocturia episodes/night based upon question 7 of the IPSS; 0–1, 2, 3, 4, and ≥ 5 nocturnal voids were reported by 425, 1931, 2160, 982, and 241 men, respectively. The dsQoL was 3.7 ± 1.2 points, the NQoL 44.5 ± 19.3 points, the NQoL-sleep 51.7 ± 20.8 points, and the NQoL-bother 48.3 ± 22.2 points (**Table 1**). All pairwise correlations between IPSS, number of nocturnal voids, dsQoL, NQoL, NQoL-sleep, and NQoL-bother were moderate to strong (Pearson correlation coefficients 0.51–0.81; **Table 2**).

**Table 1:** Clinical effects of treatment with tamsulosin OCAS.

	N	Baseline	Study end	Change
IPSS, points	5547	19.5 ± 5.9	10.1 ± 4.9	−9.4 ± 6.0
Nocturnal voids per night	5643	2.8 ± 1.0	1.3 ± 0.8	−1.4 ± 0.9
Q_max_, ml/s	1724	11.5 ± 3.7	15.8 ± 4.7	+4.3 ± 3.7
PVR, ml	4214	70 ± 49	31 ± 31	−39 ± 37
QoL, points	5675	3.7 ± 1.2	1.6 ± 1.0	−2.1 ± 1.4
NQoL-sleep, points	5558	52 ± 21	74 ± 17	+22 ± 19
NQoL-bother, points	5582	48 ± 22	77 ± 16	+29 ± 22
NQoL total, points	5638	45 ± 19	73 ± 17	+28 ± 23

Data are shown as means ± SD of baseline values and intra-individual changes.

**Table 2:** Relationship between urological symptoms and scores at baseline.

	Nocturia episodes	dsQoL	NQoL-sleep	NQoL-bother	Total NQol
Total IPSS	0.61	0.69	−0.59	−0.59	−0.57
Nocturia episodes	–	0.57	−0.53	−0.53	−0.51
dsQoL		–	−0.56	−0.60	−0.68
NQoL-sleep			–	0.81	0.65
NQoL-bother				–	0.68

Data are shown as Pearson correlation coefficients based on univariate pairwise correlations; descriptive p-values were <0.0001 in all cases. Depending on comparison, pairwise correlations are based on 5675–5762 men.

### Efficacy Results

**Figure 1:**
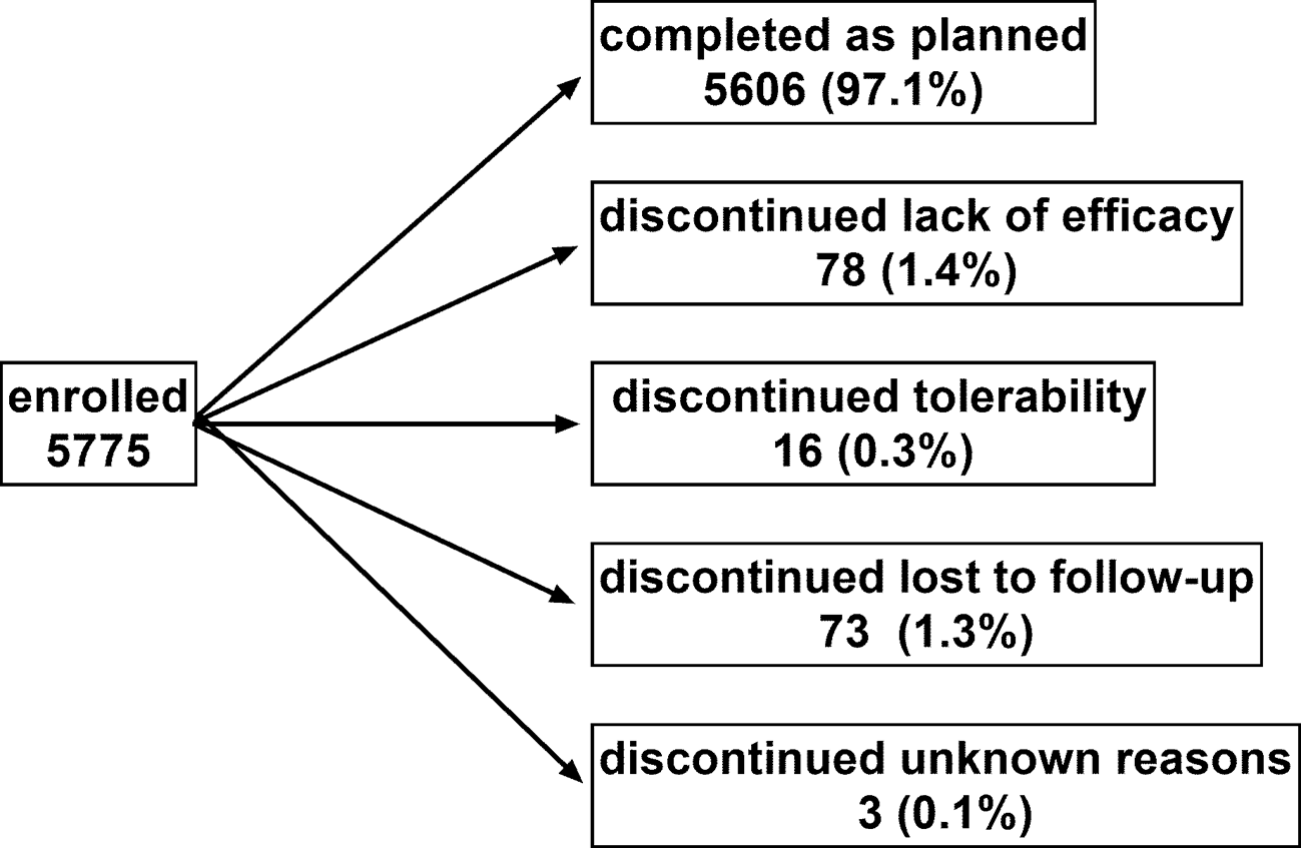
Patient disposition. Note that one man discontinued due to a combination of lack of efficacy and tolerability and was counted in each category. Sum of percentages exceeds 100 due to rounding errors.

Patient disposition is shown in **Figure 1**: 5606 men (97.1%) completed the study as planned (mean duration of observation 13.7 ± 2.7 weeks). Treatment with tamsulosin OCAS was associated with clinically meaningful improvements of total IPSS (−9.4 ± 6.0 points) including number of nocturia episodes (−1.4 ± 0.9), PVR (−38.6 ± 36.7 ml), Q_max_ (+4.3 ± 3.7 ml/s), dsQoL (+2.1 ± 1.4 points), the overall N-QoL (+28.4 ± 23.3 points), and its sleep (+21.8 ± 18.7 points) and bother domains (+28.5 ± 21.8 points; **Table 1**). All pairwise correlations between changes of IPSS, number of nocturnal voids, dsQoL, NQoL, NQoL-sleep, and NQoL-bother were moderate to strong (Pearson correlation coefficients 0.58–0.82; **Table 3**) and of comparable strength as those observed between baseline values of these parameters (**Table 2**).

**Table 3:** Relationship between changes of urological symptoms and scores between baseline and end of treatment.

	Nocturia episodes	dsQoL	NQoL-sleep	NQoL-bother	Total NQol
Total IPSS	0.68	0.75	−0.67	−0.69	−0.63
Nocturia episodes	–	0.65	−0.58	−0.61	−0.57
dsQoL		–	−0.64	−0.69	−0.70
NQoL-sleep			–	0.82	0.68
NQoL-bother				–	0.72

Data are shown as Pearson correlation coefficients based on univariate pairwise correlations; descriptive p-values were <0.0001 in all cases. Depending on comparison, pairwise correlations are based on 5431–5639 men.

### Tolerability Results

A total of 82 AEs (1.4%) were reported in 64 patients (1.1%) and led to premature discontinuation of treatment in 16 patients. Reported AEs included dizziness (n = 14), acute urinary retention/anuria (n = 6), pollakiuria/nocturia (n = 6), hypotension, and headache (n = 3 each), disturbance of orgasm/ejaculation and nausea (n = 2 each), and mild orthostatic dysregulation, dry nose, cardiac dysrhythmia, increased bladder pressure, abnormal feces, intermittent pallor of face, redness of skin, pruritus, aggravated dyspepsia, pain in urethra during micturition, insomnia, and planned transurethral resection of prostate (n = 1 each). Based on AE record forms, treatment was continued despite the observed AE in 3, reintroduced in 3 and discontinued in all other patients reporting AE. Moreover, lack of efficacy was coded as AE by the investigator in 31 patients, all leading to discontinuation. Global tolerability was rated as very good, good, satisfactory of bad by 56.0%, 37.6%, 3.0% and 0.2% of patients, respectively.

### Factors Associated With Nocturia at Baseline

**Figure 2:**
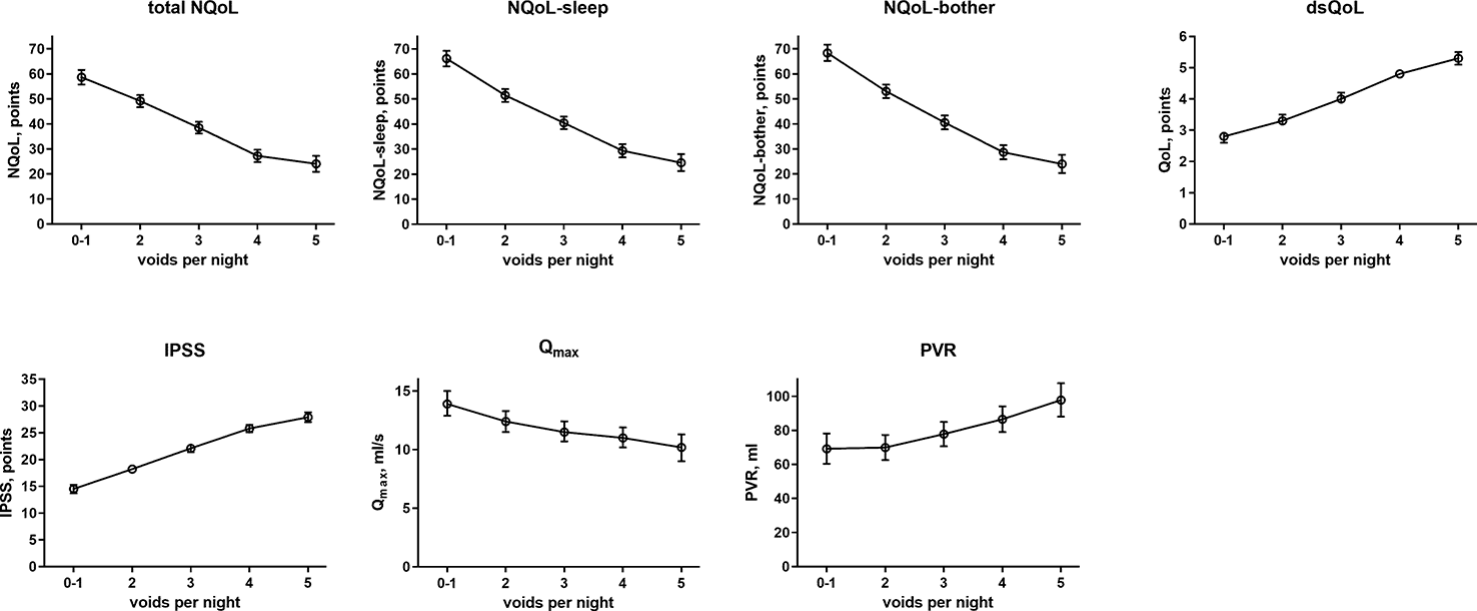
Effect sizes of number of nocturnal voids on the dependent variables NQoL, NQoL-sleep, NQoL-bother, dsQoL, IPSS, Q_max_, and PVR; effect sizes for PSA were not calculated because it was the only dependent variable with p ≥ 0.01. Data from the models that did not include nocturnal voids are shown in the **Online Supplement 1**.

**Figure 3:**
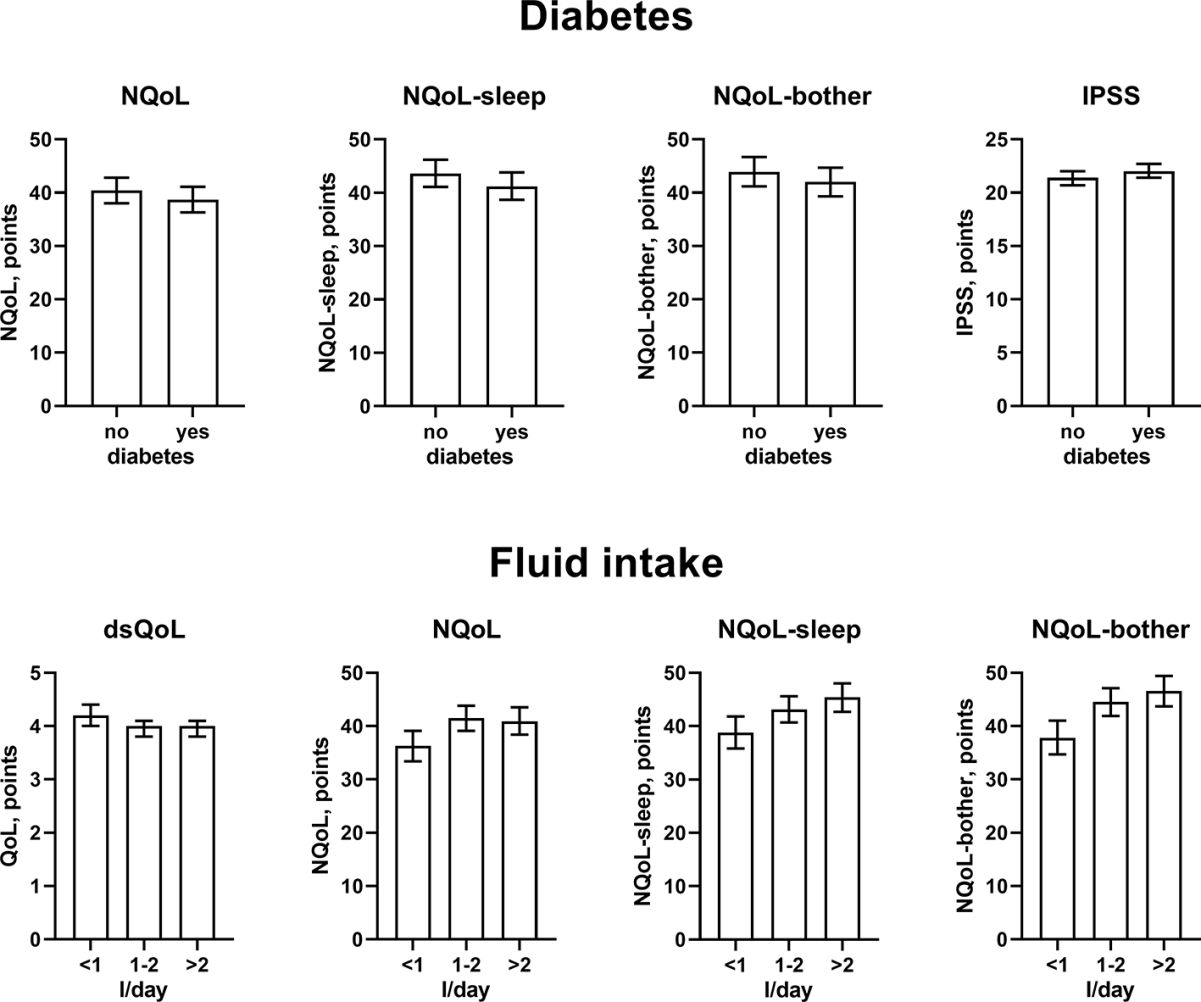
Effect sizes of presence of diabetes (upper panels) and fluid intake (lower panels) on the dependent variables NQoL, NQoL-sleep, NQoL-bother, dsQoL, and IPSS; effect sizes for other dependent variables were not calculated because p ≥ 0.01 within the model. Data are derived from the models that included number of nocturnal voids as explanatory variable; data from models not including it are shown in the **Online Supplement 1**.

The results from the general linear models with and without inclusion of number of nocturnal voids for NQoL as the dependent variable are shown in **Table 4**, all others in the **Online Supplement 1**. Number of nocturnal voids was retained in the models for all dependent variables except for PSA; effect sizes were strong for NQoL, NQoL-sleep, NQoL-bother, dsQoL, and IPSS and moderate for Q_max_ and PVR (**Figure 2**). Concomitant diabetes was retained as explanatory variable in the models for NQoL, NQoL-sleep, NQoL-bother, and IPSS and associated with a small worsening (**Figure 3**); diabetes was associated with a larger but still small worsening of dsQoL and PVR if number of nocturnal voids was not part of the model (**Online Supplement 1**). Fluid intake was retained in the models for dsQoL, NQoL, NQoL-sleep, and NQoL-bother and associated with a small to moderate worsening, particularly for NQoL and its sub-domains (**Figure 3**); fluid intake was also associated with a small worsening of the IPSS if number of nocturnal voids was not part of the model (**Online Supplement 1**). All other potentially explanatory variables were retained only in selected models and typically with small to moderate effect sizes (**Online Supplement 1**). Age was retained for Q_max_ and PVR irrespective of inclusion of nocturnal voids, but inconsistently for the other dependent variables. Body weight was only retained in one model, that for PVR if nocturnal voids were not included. Presence of congestive heart failure was included in the models for NQoL-sleep and PVR and in those for total NQoL and IPSS if nocturnal voids were not included. Sleep apnea was retained only for NQoL-sleep. Use of diuretics was retained for NQoL-sleep and that of hypnotics for IPSS and for PVR but in both cases only if nocturnal voids were not considered. Alcohol intake was retained in the models for NQoL-bother and IPSS and for dsQoL and NQoL-sleep if nocturnal voids were not considered. The only explanatory variable associated with PSA was age.

**Table 4:** Effect sizes as estimated in general linear models for associations of explanatory variables (age, weight, presence of concomitant heart failure, diabetes or sleep apnea, concomitant use of diuretics or hypnotics, fluid and alcohol intake) on the dependent variable total NQoL at baseline.

Explanatory variable	Strata	Without nocturnal void inclusion	With nocturnal void inclusion
Age	≤60 years	–	38.1 [35.6; 40.6]
	> 60 and ≤66 years	–	39.2 [36.7; 41.7]
	> 66 and ≤72 years	–	40.4 [37.9; 42.9]
	> 72 years	–	40.6 [38.1; 43.0]
Heart failure	no	39.6 [36.8; 42.4]	–
	yes	36.1 [33.2; 38.9]	–
Diabetes mellitus	no	39.7 [37.0; 42.5]	40.4 [38.0; 42.8]
	Yes	35.9 [33.2; 38.7]	38.7 [36.3; 41.1]
Fluid intake	< 1 L/day	33.3 [30.1; 36.5]	36.3 [33.4; 39.1]
	1–2 L/day	40.2 [37.6; 42.8]	41.5 [39.1; 43.8]
	> 2 L/day	40.0 [37.2; 42.8]	40.9 [38.4; 43.5]
Nocturnal voids	0–1	–	58.7 [55.8; 61.6]
	2	–	49.2 [46.7; 51.6]
	3	–	38.5 [36.2; 40.9]
	4	–	27.3 [24.8; 29.8]
	5	–	24.1 [20.9; 27.3]

Effect sizes are only shown for explanatory variables that exhibited descriptive p-values < 0.01 within the model. The models were run with (right column) and without inclusion of number of nocturnal voids (second to right column). The effect sizes are shown as means with 95% confidence intervals in the indicated strata of the explanatory variables; age and body weight strata are based on quartiles of the observed distributions. Means are adjusted for all other factors in the model. If values are not shown, they either were not part of the model (i.e. number of nocturnal voids in second to right column) or dropped because p ≥ 0.01.

### Factors Associated With Treatment-Associated Changes of Nocturia

**Figure 4:**
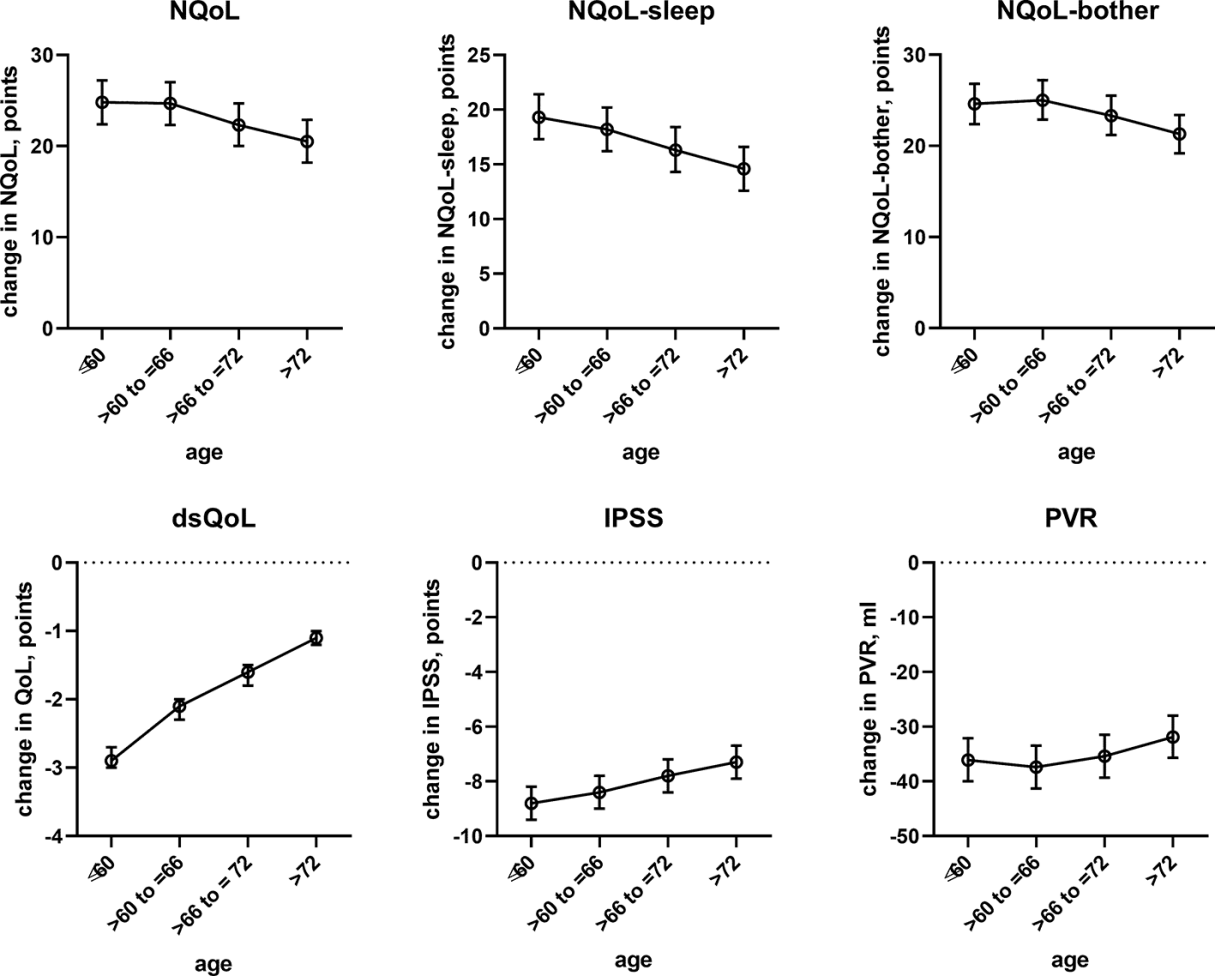
Effect sizes of age on treatment-associated changes of the dependent variables NQoL, NQoL-sleep, NQoL-bother, dsQoL, IPSS, and PVR; effect sizes for other dependent variables were not calculated because p ≥ 0.01 within the model. Data are derived from the models that included number of nocturnal voids as explanatory variable; data from models not including it are shown in the **Online Supplement 2**.

**Figure 5:**
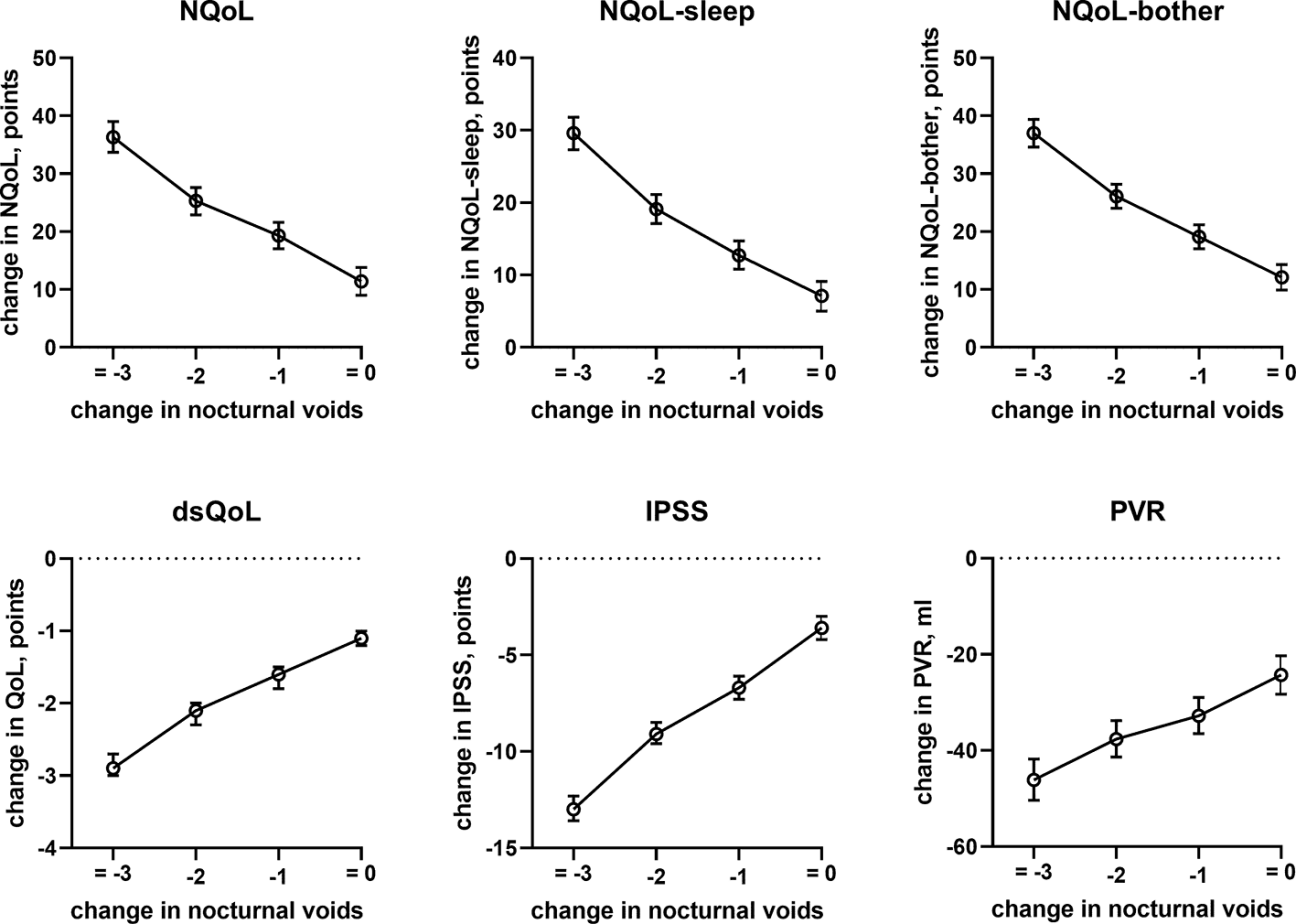
Effect sizes of change in nocturnal voids on treatment-associated changes of the dependent variables NQoL, NQoL-sleep, NQoL-bother, dsQoL, IPSS, and PVR; effect sizes for other dependent variables were not calculated because p ≥ 0.01 within the model.

**Figure 6:**
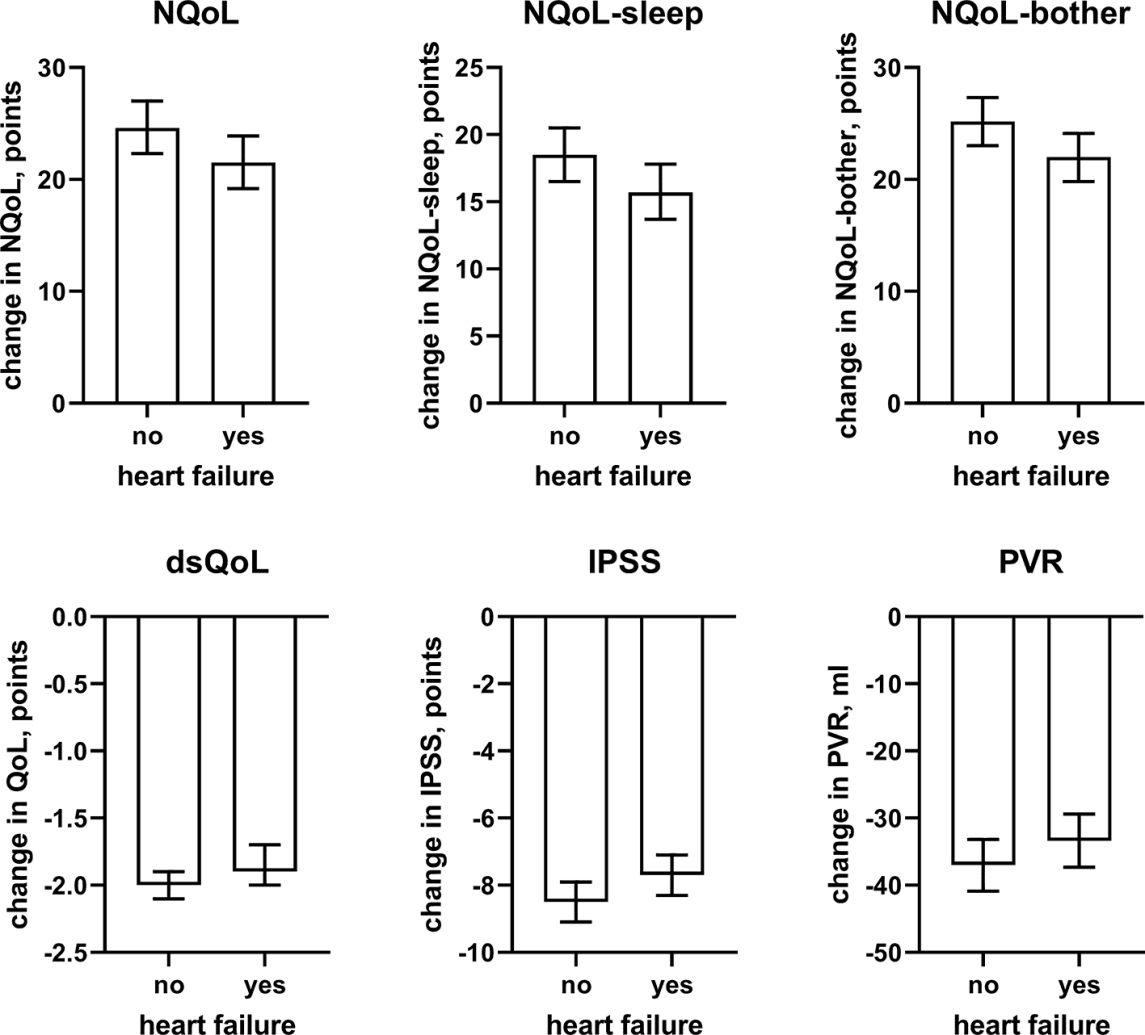
Effect sizes of presence of concomitant heart failure on the dependent variables NQoL, NQoL-sleep, NQoL-bother, dsQoL, IPSS, and PVR; effect sizes for other dependent variables were not calculated because p ≥ 0.01 within the model. Data are derived from the models that included number of nocturnal voids as explanatory variable; data from models not including it are shown in the **Online Supplement 2**.

**Figure 7:**
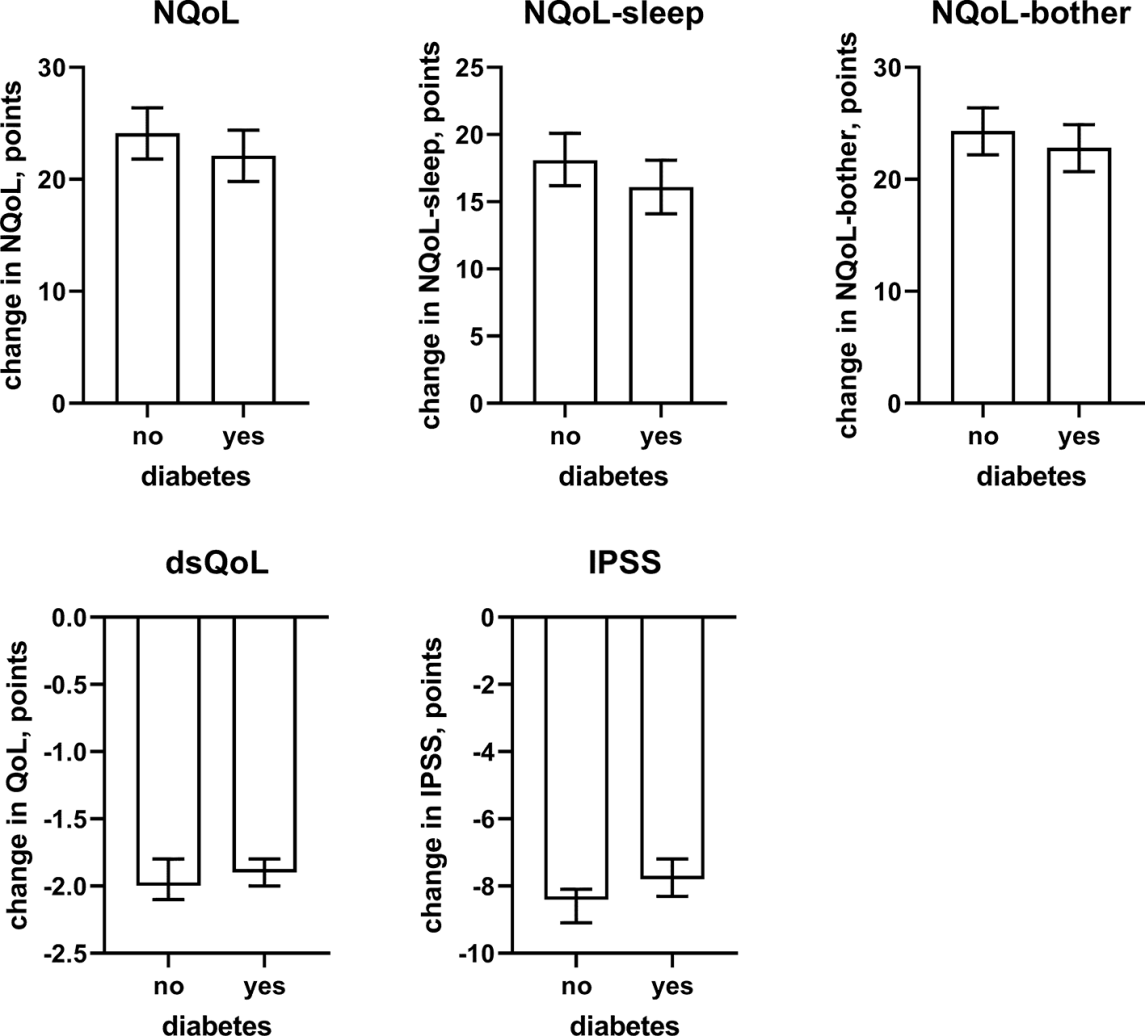
Effect sizes of presence of concomitant diabetes on the dependent variables NQoL, NQoL-sleep, NQoL-bother, dsQoL, IPSS, and PVR; effect sizes for other dependent variables were not calculated because p ≥ 0.01 within the model. Data are derived from the models that included number of nocturnal voids as explanatory variable; data from models not including it are shown in the **Online Supplement 2**.

**Figure 8:**
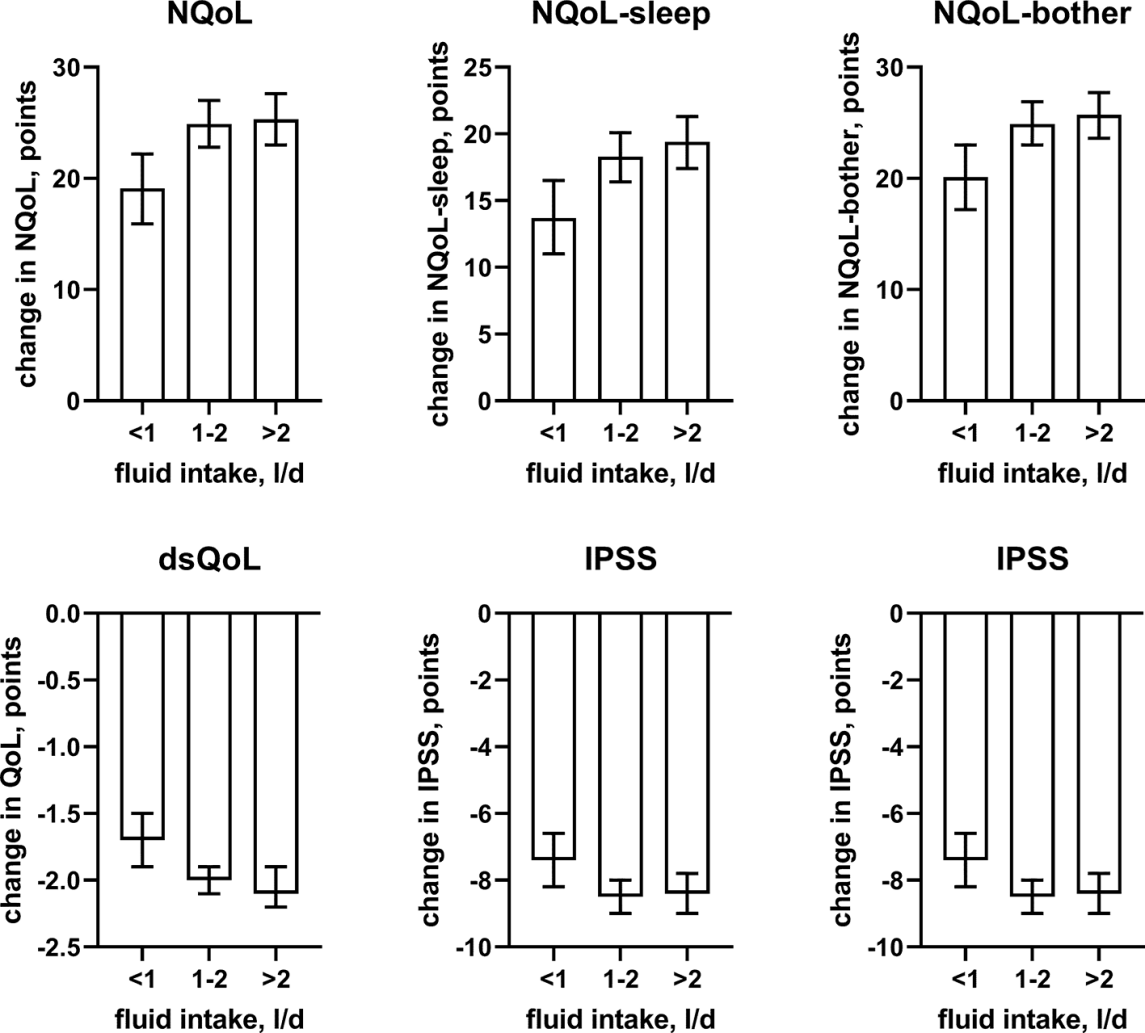
Effect sizes of fluid intake on the dependent variables NQoL, NQoL-sleep, NQoL-bother, dsQoL, IPSS, and PVR; effect sizes for other dependent variables were not calculated because p ≥ 0.01 within the model. Data are derived from the models that included number of nocturnal voids as explanatory variable; data from models not including it are shown in the **Online Supplement 2**.

The results from the general linear models with and without inclusion of changes number of nocturnal voids for treatment-associated changes of the dependent variable NQoL is shown in **Table 5**, for the other dependent variables in the **Online Supplement 2**. The results of all models share that baseline data were strongly associated with treatment-associated changes. The results differ from those exploring the baseline data (**Table 4**, **Online Supplement 1**, **Figures 2** and **3**) in several ways: First, the associations with *p* < 0.01 were the same irrespective of inclusion of number of nocturnal voids in the models assessing treatment-associated changes; the only exceptions were effect of diuretics on NQoL-sleep and of hypnotics on NQoL and NQoL-sleep, that were only retained if number of nocturnal voids was not included in the model (data from all models not including nocturnal voids are shown in the **Online Supplement 2** only). Second, concomitant heart failure and diabetes were retained in the models of all symptom scores. Third, concomitant use of hypnotics was retained in more models than for baseline data. Fourth, concomitant sleep apnea and alcohol intake were not retained in any model. Younger age was associated with greater improvements in the dsQoL, the N-QoL, and its sub-domains, the overall IPSS and the PVR; while the difference in dsQoL improvement was substantial between age groups, that of PVR was small (**Figure 4**). The change in number of nocturnal voids was also associated with greater improvement of these dependent variables but similarly so for all 6 dependent variables (**Figure 5**). The presence of concomitant heart failure was associated with moderately smaller improvements of all six dependent variables (**Figure 6**); the effect sizes for heart failure became somewhat larger when number of nocturnal voids was not part of the model (**Online Supplement 2**). The presence of concomitant diabetes was also associated with moderately smaller improvements of NQoL, NQoL-sleep, NQoL-bother, dsQoL, and IPSS but not of PVR (**Figure 7**); similar to heart failure, the effect size of diabetes became somewhat larger when number of nocturnal voids was not included in the model (**Online Supplement 2**). A medium to large fluid intake (> 1 L/d) was associated with a moderately greater improvement of the NQoL and its sub-domains and the dsQoL but only a minor improvement of IPSS (**Figure 8**).

**Table 5:** Effect sizes as estimated in general linear models for associations of explanatory variables (age, weight, presence of concomitant heart failure, diabetes or sleep apnea, concomitant use of diuretics or hypnotics, fluid and alcohol intake, respective baseline value of the dependent variable) on the treatment-associated changes of the dependent variable total NQoL.

Explanatory variable	Strata	Without nocturnal void inclusion	With nocturnal void inclusion
Age	≤60 years	21.5 [18.9; 24.0]	24.8 [22.4; 27.2]
	> 60 and ≤66 years	21.7 [19.2; 24.2]	24.7 [22.3; 27.0]
	> 66 and ≤72 years	19.2 [16.7; 21.8]	22.3 [20.0; 24.7]
	> 72 years	17.2 [14.7; 19.7]	20.5 [18.2; 22.9]
Heart failure	no	21.6 [19.1; 24.1]	24.6 [22.3; 27.0]
	yes	18.2 [15.7; 20.8]	21.5 [19.2; 23.9]
Diabetes mellitus	no	21.0 [18.5; 23.4]	24.1 [21.8; 26.4]
	yes	18.8 [16.3; 21.3]	22.1 [19.8; 24.4]
Hypnotics use	no	22.2 [20.0; 24.3]	–
	yes	17.6 [14.1; 21.1]	–
Fluid intake	< 1 L/day	13.8 [10.3; 17.2]	19.1 [15.9; 22.2]
	1–2 L/day	22.6 [20.3; 24.8]	24.9 [22.8; 27.0]
	> 2 L/day	23.4 [20.9; 25.8]	25.3 [23.0; 27.6]
Change of nocturnal voids	≤−3	–	36.3 [33.7; 39.0]
	−2	–	25.3 [22.9; 27.6]
	−1	–	19.3 [17.0; 21.6]
	≥0	–	11.4 [9.0; 13.8]

Effect sizes are only shown for explanatory variables that exhibited descriptive p-values < 0.01 within the model. Effect sizes for respective baseline values are not shown but had descriptive p-values < 0.0001 for all dependent variables. The models were run with (right column) and without inclusion of number of nocturnal voids (second to right column). The effect sizes are shown as means with 95% confidence intervals in the indicated strata of the explanatory variables; age and body weight strata are based on quartiles of the observed distributions. Means are adjusted for all other factors in the model. If values are not shown, they either were not part of the model (i.e., number of nocturnal voids in second to right column) or dropped because p ≥ 0.01.

## Discussion

Other than yielding general information on the efficacy and tolerability of tamsulosin OCAS in real-life practice, the present study performed in men with LUTS was designed to explore some of the potential non-urological factors associated with QoL and specifically nocturia-related QoL.

### Critique of Methods

Applicable laws and regulations in Germany at time of submission of our manuscript require approval by an ethical committee also for NIS. However, when our study was performed in 2005 to 2006, neither ethical committee approval nor informed patient consent was required or recommended in Germany for a NIS in which only anonymized data were communicated to the sponsor. Moreover, the study had been recorded with the German Federal Association of Statutory Health Insurance, the Kassenärztliche Bundesvereinigung, the federal regulatory body (Bundesinstitut für Arzneimittel und Medizinprodukte) and with clinicaltrials.gov (NCT02245542). As no patient was exposed to treatment for study reasons based on the non-interventional character of the study, we consider it more ethical to report our findings in the absence of ethical committee approval than to withhold them from disclosure.

Our NIS had a comparable population with regard to age, IPSS, and Q_max_ as previous NIS with the tamsulosin capsule formulation (Michel et al., 1998b; Michel et al., 2001b) or other α_1_-adrenoceptor antagonists (Lukacs et al., 1996; Höfner, 1998). Therefore, the present patient cohort is representative for men seeking treatment for LUTS.

Randomized, controlled, double-blind studies have high internal validity, but their external validity is limited because of the specific in- and exclusion criteria and because patients and physicians knowing that they are part of a study behave differently. On the other hand, a NIS typically lacks a control group and thereby has limited internal validity. However, it has greater external validity because it follows patients as they are treated in real life and lacks inclusion and exclusion criteria. Thus, the present data should not be taken as proof of efficacy but rather be seen as an indication what physicians and patients can expect during routine use of tamsulosin OCAS. However, the size of the patient cohort studied here allowed us to perform multi-variate analysis of various, possibly interacting factors that may contribute to QoL. Related to the non-interventional character of the study, our dataset comes from a large number of urologists. The advantage of this is that the data are more likely to be representative for routine care. The disadvantage is that procedures have been less standardized than they would be in a controlled study with fewer participating centers.

QoL can be evaluated in various ways. It can be assessed in a broad manner, for instance by general health-related questionnaire such as the SF-36 (Suzuki et al., 2006). However, the most frequently applied QoL indicator in the field of male LUTS is the single-item question in the IPSS, which is a 7-item scale from excellent to very bad and captures dsQoL. A QoL score specifically developed and validated for nocturia-related QoL is the NQoL (Abraham et al., 2004; Cai et al., 2006; Chartier-Kastler and Davidson, 2007). This is a 12-item questionnaire in which each item can be reported on a 5-point scale from every day/always/very much to not at all/never. Sub-domains of sleep/energy (6 items) and bother/concern (7 items; one item counted in both sub-domains) can be calculated within the NQoL. The present study has concomitantly used the dsQoL question from the IPSS and the NQoL and its two sub-domains. Of note, a worse condition is indicated by a higher score in the IPSS and its dsQoL question but by a lower score in the NQoL and its sub-domains.

Finally, it should be considered that our study is explorative and has focused on correlations. Based on the explorative character, reported p-values should be seen as descriptive only and not be interpreted as hypothesis-testing. Moreover, correlations demonstrate that parameters occur together more frequently than expected based on chance alone but do not necessarily imply a cause-effect relationship. This is highlighted by our findings that both concomitant diabetes and a smaller fluid intake were associated with a greater dsQoL and a smaller NQoL. While this would be a plausible cause-effect relationship for diabetes as causative factor, the association with smaller fluid intake, if anything, suggests adaptive behavior of the patient to cope with his nighttime symptoms. All of these aspects should be considered in the interpretation of our data.

### Factors Associated With Nocturia-related QoL at Baseline

The presence and extent of nocturia is associated with age (Yoshimura et al., 2003; Seki et al., 2008) and correlated with the overall IPSS (Cai et al., 2006). Nocturia is also associated with an impaired QoL (Ushijima et al., 2006; Seki et al., 2008; Van Dijk et al., 2010; Kupelian et al., 2012). Such correlation between nocturia and QoL has not only been found men with LUTS but also in cancer patients undergoing chemotherapy (Batista-Miranda et al., 2007). The associations between number of nocturia episodes and overall IPSS or dsQoL were confirmed in the present study (**Table 2**, **Figure 2**); moreover, we show that number of nocturia episodes is also associated with a lower Q_max_ and a greater PVR—although the effect appears smaller than that for QoL scores. As number of nocturia episodes was not associated with PSA in our study and PSA is a proxy for prostate size (Roehrborn et al., 1999; Mochtar et al., 2003), this implies that prostate size is not associated with number of nocturia episodes either. A lack of correlation between of prostate size and number of nocturnal voids was also reported by others in direct comparison of the two parameters (Seki et al., 2008).

Against this background, our study focused less on nocturia episodes themselves and rather on factors associated with nocturia-related QoL as captured in the NQoL and dsQoL, particularly with regard to non-urological contributions. A previous study in 109 men has reported an almost linear relationship between number of nocturnal voids and NQoL-sleep, but that relationship flattened if >5 nocturnal episodes were present (Cai et al., 2006). Expanding these findings in a much larger cohort, we have found that number of nocturia episodes makes the strongest and most consistent statistical contribution to QoL among the explanatory variables explored here. We also found that concomitant diabetes is associated with a smaller NQoL and a greater dsQoL (**Figure 3**), which is not surprising since diabetes is a known cause of nocturia. However, effect sizes were only moderate at best compared to those of nocturia itself, particularly in the models including number of nocturnal voids. Effect sizes were larger in the models not including number of nocturnal voids as explanatory variable (**Table 4** and **Online Supplement 1**), indicating that the effect of diabetes on nocturia-related QoL is largely driven by its effect on nocturia itself. The most likely explanation for the relatively small impact of concomitant diabetes is that we have studied a male LUTS population, i.e. a group of patients that had been selected based on their LUTS. A small effect size was also observed for fluid intake, but it was not a greater (as expected on the diuresis hypothesis) but rather a smaller fluid intake that was associated with poorer QoL. The most likely explanation for this is that the cause-effect relationship, if any, is opposite, i.e. that men with nocturia and a high NQoL drink less in an attempt to manage their LUTS. Interestingly, other suspected explanatory factors such as presence of heart failure or sleep apnea, use of diuretics or alcohol consumption had only small and inconsistent associations with the various symptom scores. The small effect sizes for all non-urological candidate factors may at least partly be explained by the fact that our analysis was not population-based but performed in a cohort of patients seeking treatment for male LUTS. Thus, it is not surprising that non-urological causes of nocturia contribute to the corresponding QoL scores in a limited way only.

### Clinical Treatment Outcomes

Other than one large phase III study (Chapple et al., 2005), only few and largely small studies have investigated the efficacy and safety of the OCAS formulation of tamsulosin (Van Kerrebroeck et al., 2013). The improvements of the IPSS and the incidence and type of AEs in the present study with tamsulosin OCAS were comparable to those in previous NIS with the capsule formulation of tamsulosin (Michel et al., 1998b; Michel et al., 2001b). Specifically, the reduction in number of nocturnal voids is very similar to that in previous NIS with the capsule formulation of tamsulosin (−1.4 vs. −1.3 episodes) (Schneider et al., 2009). The similar extent of improvement in the present study with tamsulosin OCAS and previous NIS with the capsule formulation of tamsulosin is line with findings from a double-blind direct comparative study between the two formulations also showing comparable efficacy of both formulations (Chapple et al., 2005). However, the improvements in male LUTS were greater than typically reported in placebo-controlled studies (Milani and Djavan, 2005). NIS in men with LUTS differ from randomized, placebo-controlled studies in two ways: they are not performed according to Good Clinical Practice guidelines and they lack a placebo-controlled run-in period (Michel and Goepel, 2000). We do not consider the former a likely explanation because the efficacy in the NIS with the capsule formulation of tamsulosin and in the present study with tamsulosin OCAS is also in line with findings from an open-label phase IIIb study performed according to Good Clinical Practice guidelines with the capsule formulation of tamsulosin (Michel et al., 2001a). However, we consider the latter a likely explanation because some improvement of LUTS already occurs in the placebo-controlled run-in period of randomized controlled studies (Chapple et al., 1996); unfortunately, the extent of the improvement prior to randomization is rarely reported.

### Factors Associated With Treatment-Associated Improvement of Nocturia-Related QoL

In line with previous reports (Michel et al., 1998a), age had only a minor impact on the treatment-associated improvement of the IPSS; however, age is associated with nocturia (Seki et al., 2008). We now extend these findings by demonstrating a minor impact of age on improvements of PVR. In contrast, we found that age has a major impact on treatment-associated improvements of the NQoL and its sub-domains and the dsQoL (**Figure 4**). Given that improvements of the IPSS in this and previous studies (Michel et al., 1998a) exhibit only a minor age component, these data indicate that QoL improves less in the elderly for a comparable change in symptoms.

In line with the association between number of nocturnal voids in the present and a previous study (Cai et al., 2006), we also found that change in number of nocturnal voids has a major impact on improvements of the NQoL and its sub-domains, the dsQOL, the IPSS, and even the PVR (**Figure 5**). While concomitant heart failure or diabetes were associated with smaller improvements of all QoL scores and the IPSS (**Figures 6** and **7**), the magnitude of the effect sizes was small compared to that of changes in number of nocturnal voids after adjustment for number of nocturnal voids. However, estimated effect sizes of heart failure and diabetes were larger if number of nocturnal voids was not part of the model (**Table 5** and **Online Supplement 2**), indicating that the impact of heart failure and diabetes on the NQoL is largely driven by its effect on nocturia. The overall limited effect size of concomitant diabetes or heart failure is most likely explained by the fact that our study population had been selected based on LUTS. In contrast, nocturnal polyuria has been reported to be present in more than 75% of patients considered for treatment with tamsulosin (Xue et al., 2018). This raises the question whether tamsulosin may affect nocturnal polyuria. While one study found that treatment with tamsulosin reduced nighttime urine volume (Kojima et al., 2012), another study did not confirm this (Xue et al., 2018). Both hypotheses have some degree of pathophysiological plausibility: on the one hand, α_1_-adrenoceptor antagonism can reduce nerve stimulation-induced sodium retention in rat (Smyth et al., 1985). On the other hand, α_1_-adrenoceptor did not affect vasopressin effects on cortical collecting ducts in rats (Dillingham and Anderson, 1989) and acute treatment with the α_1_-adrenoceptor antagonist bunazosin did not affect circulating levels of vasopressin in humans (Tomiyama et al., 1992). Additional studies are required to firmly establish whether α_1_-adrenoceptor antagonism affects diuresis and to establish the relative roles of an enlarged prostate, nocturnal polyuria, diabetes, and heart failure in the general population.

## Conclusions

We conclude that number of nocturnal voids is the key driver of nocturia-related QoL in men seeking treatment for their LUTS. Known causes of nocturia, such as heart failure and diabetes or intake of alcohol or use of diuretics, contribute less to nocturia-related QoL. The contribution of the non-urological factors appears largely driven by their effect on number of nocturia episodes, but a small effect independent of the number of nocturnal voids was also detected and may reflect the general effect of comorbidity on QoL. These findings do not support the hypothesis that limited efficacy of α_1_-adrenoceptor antagonists against nocturia as part of male LUTS is primarily explained by non-urological causes of nocturia. Given the important impact of nocturia on QoL, morbidity, and mortality, more effective treatments for nocturia in the male LUTS population are needed.

## Data Availability Statement

The datasets generated for this study are available on request to the corresponding author.

## Ethics Statement

Ethical review and approval was not required for the study on human participants in accordance with the local legislation and institutional requirements. Written informed consent for participation was not required for this study in accordance with the national legislation and the institutional requirements.

## Author Contributions

All authors have jointly developed the statistical analysis plan. HS performed the statistical analysis. MM drafted the manuscript. All authors have made substantial comments to the manuscript draft and approved the final version.

## Funding

This work was supported in part by grant Mi 294/10-1 from the Deutsche Forschungsgemeinschaft to MM. The underlying study and the fee for open-access publication were funded by Boehringer Ingelheim.

## Conflict of Interest

In the field of urology, MM has been a consultant and/or lecturer in the past 5 years to Apogepha, Astellas, Dr. Willmar Schwabe, Ferring, GSK, Recordati, and Velicept; he was a consultant to Boehringer Ingelheim at the time the present study was designed; he also has been an employee of Boehringer Ingelheim (2011–2016) and is a shareholder of Velicept. LM is an employee of Boehringer Ingelheim and HS is a past employee (until 2015) and present consultant of Boehringer Ingelheim.

The remaining author declares that the research was conducted in the absence of any commercial or financial relationships that could be construed as a potential conflict of interest.

## Abbreviations

AE, adverse event; dsQoL, disease-specific quality of life score; IPSS, International Prostate Symptom Score; LUTS, lower urinary tract symptom; NIS, non-interventional study; NQoL, nocturia quality of life score; OCAS, oral controlled absorption system; PVR, post-voiding residual urine; PSA, prostate-specific antigen; QoL, quality of life.
