# Supplementary material for: Factors Associated With Nocturia-Related Quality of Life in Men With Lower Urinary Tract Symptoms and Treated With Tamsulosin Oral Controlled Absorption System in a Non-Interventional Study
Source: Front Pharmacol. 2020 Jun 4;11:816. doi: 10.3389/fphar.2020.00816 (PMC7287125; doi:10.3389/fphar.2020.00816)
Supplement: Supplementary file 1 [file DataSheet_1.docx]

**Online Supplement 1 to**

**Factors associated with disease-specific and nocturia-related quality of life in men with lower urinary tract symptoms and treated with tamsulosin oral-controlled absorption system**

**Martin C. Michel, Helmut Schumacher, Ludwig Mehlburger, Jean J.M.C.H. de la Rosette**

The tables below show effect sizes as estimated in general linear models for associations of explanatory variables (age, weight, presence of concomitant heart failure, diabetes or sleep apnea, concomitant use of diuretics or hypnotics, fluid and alcohol intake) on the dependent variables NQoL sleep, NQoL bother, dsQoL, total IPSS, Q_max_, PVR and PSA. Effect sizes are only shown for explanatory variables that exhibited descriptive p-values < 0.01 within the model. The models were run with (right column) and without inclusion of number of nocturnal voids (second to right column). The effect sizes are shown as means with 95% confidence intervals in the indicated strata of the explanatory variables; age and body weight strata are based on quartiles of the observed distributions. Means are adjusted for all other factors in the model. If values are not shown, they either were not part of the model (i.e. number of nocturnal voids in second to right column) or dropped because p ≥ 0.01.

## Dependent variable: NQoL-sleep

| Explanatory variable | Strata | Without nocturnal void inclusion | With nocturnal  void inclusion |
| --- | --- | --- | --- |
| Heart failure | no | 42.2 [39.3; 45.2] | 43.6 [41.0; 46.2] |
|  | yes | 37.7 [34.7; 40.7] | 41.3 [38.6; 43.9] |
| Diabetes mellitus | no | 42.3 [39.4; 45.3] | 43.6 [41.1; 46.2] |
|  | Yes | 37.6 [34.7; 40.5] | 41.2 [38.7; 43.8] |
| Sleep apnea | No | 45.2 [43.1; 47.2] | 47.1 [45.3; 48.9] |
|  | yes | 34.7 [30.0; 39.5] | 37.7 [33.6; 41.9] |
| Diuretics use | No | 41.5 [38.6; 44.4] | - |
|  | Yes | 38.4 [35.3; 41.5] | - |
| Fluid intake | <1 l/day | 35.0 [31.6; 38.5] | 38.8 [35.8; 41.8] |
|  | 1-2 l/day | 41.2 [38.4; 44.0] | 43.1 [40.7; 45.6] |
|  | >2 l/day | 43.6 [40.6; 46.7] | 45.4 [42.7; 48.0] |
| Alcohol intake | No | 38.7 [35.9; 41.6] | - |
|  | Yes | 41.2 [38.2; 44.1] | - |
| Nocturnal voids | 0-1 | - | 66.2 [63.1; 69.3] |
|  | 2 | - | 51.5 [48.9; 54.0] |
|  | 3 | - | 40.5 [38.0; 43.0] |
|  | 4 | - | 29.4 [26.7; 32.0] |
|  | 5 | - | 24.6 [21.2; 28.0] |

## Dependent variable: NQoL-bother

| Explanatory variable | Strata | Without nocturnal void inclusion | With nocturnal  void inclusion |
| --- | --- | --- | --- |
| Age | ≤60 years | - | 41.6 [38.7; 44.4] |
|  | >60 and ≤66 years | - | 41.5 [38.7; 44.3] |
|  | >66 and ≤ 72 years | - | 43.8 [41.0; 46.6] |
|  | > 72 years | - | 45.0 [42.2; 47.8] |
| Diabetes mellitus | no | 42.5 [39.4; 45.7] | 43.9 [41.2; 46.7] |
|  | Yes | 38.0 [34.9; 41.1] | 42.0 [39.3; 44.7] |
| Fluid intake | <1 l/day | 33.8 [30.1; 37.5] | 37.8 [34.7; 41.0] |
|  | 1-2 l/day | 42.3 [39.3; 45.3] | 44.5 [41.9; 47.1] |
|  | >2 l/day | 44.7 [41.4; 48.0] | 46.6 [43.7; 49.4] |
| Alcohol intake | No | 38.6 [35.5; 41.6] | 41.6 [38.9; 44.3] |
|  | Yes | 41.9 [38.8; 45.1] | 44.3 [41.6; 47.1] |
| Nocturnal voids | 0-1 | - | 68.4 [65.2; 71.7] |
|  | 2 | - | 53.1 [50.4; 55.8] |
|  | 3 | - | 40.6 [37.9; 43.4] |
|  | 4 | - | 28.7 [25.9; 31.5] |
|  | 5 | - | 24.0 [20.4; 27.7] |

## Dependent variable: dsQoL, points

| Explanatory variable | Strata | Without nocturnal void inclusion | With nocturnal  void inclusion |
| --- | --- | --- | --- |
| Age | ≤60 years | - | 4.1 [4.0; 4.3] |
|  | >60 and ≤66 years | - | 4.1 [4.0; 4.2] |
|  | >66 and ≤ 72 years | - | 4.0 [3.9; 4.2] |
|  | > 72 years | - | 3.9 [3.8; 4.1] |
| Diabetes mellitus | no | 4.0 [3.8; 4.2] | - |
|  | Yes | 4.2 [4.1; 4.4] | - |
| Fluid intake | <1 l/day | 4.4 [4.2; 4.5] | 4.2 [4.0; 4.4] |
|  | 1-2 l/day | 4.0 [3.8; 4.2] | 4.0 [3.8; 4.1] |
|  | >2 l/day | 4.0 [3.8; 4.2] | 4.0 [3.8; 4.1] |
| Alcohol intake | No | 4.2 [4.0; 4.3] | - |
|  | Yes | 4.1 [3.9; 4.2] | - |
| Nocturnal voids | 0-1 | - | 2.8 [2.6; 2.9] |
|  | 2 | - | 3.3 [3.2; 3.5] |
|  | 3 | - | 4.0 [3.9; 4.2] |
|  | 4 | - | 4.8 [4.7; 4.9] |
|  | 5 | - | 5.3 [5.1; 5.5] |

## Dependent variable: total IPSS, points

| Explanatory variable | Strata | Without nocturnal void inclusion | With nocturnal  void inclusion |
| --- | --- | --- | --- |
| Age | ≤60 years | 21.7 [20.9; 22.6] | - |
|  | >60 and ≤66 years | 22.4 [21.5; 23.2] | - |
|  | >66 and ≤ 72 years | 22.3 [21.5; 23.2] | - |
|  | > 72 years | 22.7 [21.9; 23.5] | - |
| Heart failure | no | 21.7 [20.8; 22.5] | - |
|  | yes | 22.9 [22.0; 23.7] | - |
| Diabetes mellitus | no | 21.6 [20.7; 22.4] | 21.4 [20.7; 22.0] |
|  | Yes | 23.0 [22.2; 23.8] | 22.0 [21.4; 22.7] |
| Hypnotics use | No | 21.3 [20.6; 22.0] | 21.0 [20.5; 21.6] |
|  | Yes | 23.2 [22.1; 24.4] | 22.3 [21.4; 23.3] |
| Fluid intake | <1 l/day | 23.0 [22.1; 24.0] | - |
|  | 1-2 l/day | 22.0 [21.2; 22.8] | - |
|  | >2 l/day | 21.8 [20.9; 22.6] | - |
| Alcohol intake | No | 22.6 [21.8; 23.4] | 22.0 [21.3; 22.6] |
|  | Yes | 21.9 [21.1; 22.7] | 21.4 [20.8; 22.1] |
| Nocturnal voids | 0-1 | - | 14.5 [13.7; 15.3] |
|  | 2 | - | 18.2 [17.6; 18.9] |
|  | 3 | - | 22.1 [21.4; 22.7] |
|  | 4 | - | 25.8 [25.1; 26.5] |
|  | 5 | - | 27.9 [27.0; 28.8] |

## Dependent variable: Q_max_, ml/s

| Explanatory variable | Strata | Without nocturnal void inclusion | With nocturnal  void inclusion |
| --- | --- | --- | --- |
| Age | ≤60 years | 12.6 [11.7; 13.4] | 12.7 [11.8; 13.5] |
|  | >60 and ≤66 years | 11.7 [10.8; 12.6] | 12.0 [11.1; 12.9] |
|  | >66 and ≤ 72 years | 10.9 [10.1; 11.8] | 11.3 [10.4; 12.2] |
|  | > 72 years | 10.9 [10.0; 11.8] | 11.3 [10.4; 12.2] |
| Nocturnal voids | 0-1 | - | 13.9 [12.9; 15.0] |
|  | 2 | - | 12.4 [11.5; 13.3] |
|  | 3 | - | 11.5 [10.7; 12.4] |
|  | 4 | - | 11.0 [10.2; 11.9] |
|  | 5 | - | 10.2 [9.0; 11.3] |

## Dependent variable: PVR, ml

| Explanatory variable | Strata | Without nocturnal void inclusion | With nocturnal  void inclusion |
| --- | --- | --- | --- |
| Age | ≤60 years | 73.0 [65.5; 80.6] | 75.2 [67.6; 82.8] |
|  | >60 and ≤66 years | 77.6 [70.2; 85.0] | 78.8 [71.3; 86.3] |
|  | >66 and ≤72 years | 82.0 [74.5; 89.5] | 82.4 [74.9; 90.0] |
|  | > 72 years | 84.8 [77.5; 92.2] | 84.4 [77.0; 91.8] |
| Weight | ≤76.5 kg | 79.5 [72.0; 87.0] | - |
|  | >76.5 and ≤82.0 kg | 75.4 [67.9; 82.8] | - |
|  | >82.0 and ≤88.5 kg | 79.9 [72.4; 87.4] | - |
|  | >88.5 kg | 82.7 [75.5; 90.0] | - |
| Heart failure | no | 21.7 [20.8; 22.5] | 76.9 [69.5; 84.3] |
|  | yes | 22.9 [22.0; 23.7] | 83.5 [76.0; 91.1] |
| Diabetes mellitus | no | 21.6 [20.7; 22.4] | - |
|  | Yes | 23.0 [22.2; 23.8] | - |
| Hypnotics use | No | 21.3 [20.6; 22.0] | - |
|  | Yes | 23.2 [22.1; 24.4] | - |
| Nocturnal voids | 0-1 | - | 69.2 [60.4; 78.1] |
|  | 2 | - | 69.9 [62.5; 77.2] |
|  | 3 | - | 77.8 [70.6; 84.9] |
|  | 4 | - | 86.5 [79.0; 94.0] |
|  | 5 | - | 97.8 [88.0; 107.7] |

## Dependent variable: PSA, ng/ml

| Explanatory variable | Strata | Without nocturnal void inclusion | With nocturnal  void inclusion |
| --- | --- | --- | --- |
| Age | ≤60 years | 2.7 [2.3; 3.0] | 2.6 [2.3; 3.0] |
|  | >60 and ≤66 years | 3.0 [2.6; 3.3] | 2.9 [2.6; 3.3] |
|  | >66 and ≤72 years | 3.1 [2.8; 3.5] | 3.1 [2.7; 3.4] |
|  | > 72 years | 3.6 [3.3; 4.0] | 3.6 [3.2; 3.9] |
